# Xenofree generation of limbal stem cells for ocular surface advanced cell therapy

**DOI:** 10.1186/s13287-019-1501-9

**Published:** 2019-12-04

**Authors:** Nuria Nieto-Nicolau, Eva M. Martínez-Conesa, Alba M. Velasco-García, Caterina Aloy-Reverté, Anna Vilarrodona, Ricardo P. Casaroli-Marano

**Affiliations:** 1 Barcelona Tissue Bank, Banc de Sang i Teixits (BST), Barcelona, Spain; 2 Institute of Biomedical Research (IIB-Sant Pau; SGR1113), Barcelona, Spain; 3 Department of Surgery, School of Medicine & Hospital Clinic de Barcelona, University of Barcelona, Barcelona, Spain

**Keywords:** Cultured limbal stem cells transplantation, XSHEM, CnT07, Processed lipoaspirate cells, 3T3, Clinical grade, Limbal stem cells deficiency

## Abstract

**Background:**

Limbal stem cells (LSC) sustain the corneal integrity and homeostasis. LSC deficiency (LSCD) leads to loss of corneal transparency and blindness. A clinical approach to treat unilateral LSCD comprises autologous cultured limbal epithelial stem cell transplantation (CLET). CLET uses xenobiotic culture systems with potential zoonotic transmission risks, and regulatory guidelines make necessary to find xenofree alternatives.

**Methods:**

We compared two xenofree clinical grade media and two feeder layers. We used CnT07, a defined commercial medium for keratinocytes, and a modified xenofree supplemented hormonal epithelial medium with human serum (XSHEM). Optimal formulation was used to compare two feeder layers: the gold standard 3T3 murine fibroblasts and human processed lipoaspirate cells (PLA). We tested the expressions of ΔNp63α and cytokeratin 3 and 12 by qPCR and immunofluorescence. Morphology, viability, clonogenicity, proliferation, and cell growth assays were carried out. We also evaluated interleukin 6 (IL-6) and stromal-derived factor 1 (SDF-1) by qPCR and ELISA.

**Results:**

XSHEM maintained better LSC culture viability and morphology than CnT07. Irradiated PLA feeder cells improved the undifferentiated state of LSC and enhanced their growth and clonogenicity stimulating IL-6 secretion and SDF-1 expression, as well as increased proliferation and cell growth when compared with irradiated 3T3 feeder cells.

**Conclusions:**

The combination of XSHEM and PLA feeder cells efficiently sustained LSC xenofree cultures for clinical application. Moreover, PLA feeder layers were able to improve the LSC potential characteristics. Our results would have direct clinical application in CLET for advanced therapy.

**Graphical abstract:**

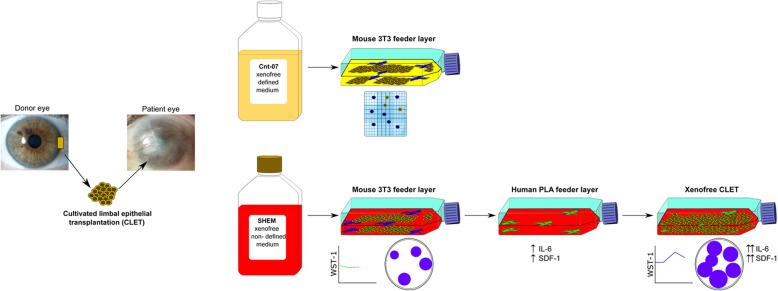

## Background

The transparency and the integrity of the cornea are maintained by a subset of stem cells located at the epithelial basal layer of the limbus, an anatomic circumferential area that separates the transparent cornea from the conjunctiva [1]. These stem cells are called limbal stem cells (LSC), and they are defined by their small size, high nucleus-to-cytoplasm ratio [1, 2], and positivity for the putative stemness marker ΔNp63α [2, 3] as well as negativity for corneal epithelial differentiation markers cytokeratin (CK) 12 and CK3 [2]. The loss of LSC produces new vessel formation, corneal conjunctivalization, and scarring, leading to corneal blindness [4]. Limbal stem cell deficiency (LSCD) can be caused by chemical, traumatic, and infectious insults and also by genetic etiologies [4]. Its prevalence is increasing, due to the use of corrosive cleaners in the household field [5]. Approximately, LSCD affects approximately 10 million people worldwide [6, 7].

Although the technique for the treatment of unilateral LSCD has evolved with time, the current gold standard treatment for unilateral LSCD is cultured limbal epithelial stem cell transplantation (CLET) [8]. For CLET, the LSC can be cultured by explant or cell suspension systems [9]. In the first system, a small biopsy of the healthy limbus is seeded on amniotic membrane. The cells grow, and the sheet is transplanted onto the damaged eye. In the cell suspension approach, LSC obtained from a minimally invasive limbal biopsy are enzymatically disaggregated and ex vivo expanded on an inactivated feeder layer of 3T3 murine fibroblasts until sub-confluence. Then, the cells are detached and seeded on a biocompatible carrier for transplantation [8, 9]. This approach is more advantageous than explant systems, since it reduces the risk of contamination of the culture by other limbal cells (such as stromal fibroblasts) [10] and increases the amount of cells that can be obtained due to higher proliferation rates [11–13]. Moreover, the cell suspension cultures are an optimized option since they are more enriched in stem cell progenies than explant culture methods [10, 12, 13] leading to improved outcomes [14].

Since the first use of CLET for human LSCD treatment in 1997 [15], xeno-products have been used for the ex vivo expansion of LSC both in explant and cell suspension systems [16]. However, European regulatory guidelines [17, 18] for the safety and quality of human tissues and cells encourage the implementation of standard operating procedures to prevent the use of xenogeneic compounds and the potential associated contamination. Although the use of xeno-products supposes a risk for human health by virus, prions, and zoonoses transmission [19], little research has been made to avoid xenobiotics during LSC culture in suspension systems [20–22]. Besides, traditional LSC media for clinical application contain cholera toxin [15, 23], increasing the risk of disease transmission. So, substitution of the LSC culture medium containing serum and xenobiotics from animal origin, along with the replacement of the feeder layer of murine 3T3 fibroblasts by xenofree alternatives, is of utmost significance.

To study clinical grade xenofree alternatives that could serve to maintain LSC cultures for cell therapy in humans, we tested two xenofree media and two feeder layer approaches. We compared a defined commercial medium, designed to sustain keratinocyte growth, with a supplemented hormonal epithelial medium complemented with human serum (XSHEM). Then, we performed a comparison between the gold standard murine 3T3 fibroblasts [16] and human processed lipoaspirate cells (PLA) as a feeder layer for LSC culture growth. Our results propose a clinical grade alternative free of xenobiotics to sustain optimal LSC culture growth with direct clinical application for advanced therapy.

## Materials and methods

### Cell culture of feeder layers

Murine 3T3 Swiss Albino fibroblasts were obtained from Kerafast (3T3-J2, EF3003). Cells were cultured with DMEM 4.5 g/l (Thermo Scientific, MA, USA) supplemented with 10% FCS and 1% antibiotics. Human processed lipoaspirate cells (PLA) from fresh human lipoaspirates were collected from healthy donors, during plastic liposuction procedures, in planned lipoaspiration surgeries. PLA were obtained by stromal vascular fraction isolation, and cultured and characterized as previously described [24]. PLA cells accomplished the criteria for mesenchymal stem cell characterization (data not shown) [25]. Then, both 3T3 and PLA cells were inactivated by irradiation with 6000 rads. After this, cells were plated onto culture dishes at 2 × 10^4^ cells/cm^2^ for feeder-layer use or downstream experiments.

### Human LSC and corneal epithelial cells

Cadaveric adult human limbal tissues from six different donors were obtained from the Barcelona Tissue Bank (BTB-BST, Barcelona, Spain; http://www.bancsang.net/en_index/). LSC were isolated as previously described [26, 27]. LSC from each donor were equally divided and cultured until sub-confluence with xenofree supplemented hormonal epithelial media (XSHEM) or CnT07 medium (CellnTec, Bern, Switzerland) on 3T3 or PLA feeder layers that were seeded 24 h before. XSHEM composition consisted of the following: Dulbecco’s modified Eagle’s medium/Ham’s and F-12 (2:1 vol:vol) (DMEM/F12; Invitrogen, Carlsbad, CA) supplemented with 2 mM l-glutamine (Lonza, Verviers, Belgium), 5 μg/ml human insulin (Sigma Aldrich, Munich, Germany), 10 ng/ml human epidermal growth factor (hEGF, Sigma-Aldrich), 0.5% dimethyl sulfoxide (DMSO, Sigma-Aldrich), 0.4 μg/ml hydrocortisone (Sigma-Aldrich), 2 nM triiodothyronine (Sigma-Aldrich), 0.18 mM adenine (Sigma-Aldrich), and 10% Human AB Serum (Corning, Manassas, VA). After the isolation passage, cells were used for downstream applications. Human corneal epithelial cells (CO) were obtained by mechanical scrapping of the central corneal epithelium of five different donors, avoiding the perilimbal region, and used as control for qPCR experiments.

### Colony-forming assay (CFA) and doubling population time (DPT)

For CFA determination, LSC that were previously cultured on 3T3 or PLA feeder layers were seeded in 35-mm-diameter plates and cultured for 14 days [28] with 3T3 feeder-layer support. Colonies were fixed and stained with 0.5% crystal violet in methanol. Analysis was performed according to previous criteria and presented as a percentage after applying the previously described formula [29]. The diameter of each colony was measured using ImageJ software [30]*.* DPT was calculated as described elsewhere [31].

### Immunofluorescence (IF)

Cells of each experimental group (5 × 10^5^) were added to ThinPrep® PreservCyt solution (Hologic Iberia SL, Barcelona, Spain) for fixation and preservation. Cells were then transferred to slides using ThinPrep 3000 processor (Hologic), which allowed the cells to be seeded in a single plane without forming clumps. Slides were preserved in methanol until use, permeabilized, blocked, and then incubated with primary antibodies. After several washes in 100 mM PBS solution, proper secondary antibody was added for 60 min at 37 °C in a humidified chamber. The antibodies and concentrations used are detailed in Additional file 2: Table S1. Cells were observed in an epifluorescence microscope (BX61; Olympus R-FTL-T; Olympus America Inc., Center Valley, PA), coupled with a program for digital image acquisition (Olympus DP Controller Program). Images were processed with ImageJ software [30].

### mRNA extraction and quantitative polymerase chain reaction (qPCR) analysis

Total RNA was extracted from co-cultures of LSC with either 3T3 or PLA feeder layers or from monocultures of the feeder layers at the last day of LSC culture. The extraction was performed using RNA Purelink Mini Kit (Ambion, Invitrogen), following the manufacturer’s instructions. The RNA concentration was measured using NanoDrop lite spectrophotometer (Thermo Scientific). RNA (1 μg) was reverse-transcribed using Superscript III (Invitrogen) according to the manufacturer’s instructions. Then, cDNA (1 μl) was used for qPCR in a final volume of 18 μl with Lightcycler 480 Sybr Green I Master (Roche, Barcelona, Spain) and a 0.2-μM primer concentration. The qPCR was performed using Lightcycler 480 II (Roche) hardware and software. The expression level of target genes was normalized to internal 18s (rrn18s, TATAA Biocenter, Sweden) and represented as relative expression using 2^-ΔΔCt^ formula. The sequences and annealing temperatures of PCR primers are listed in Additional file 2: Table S2.

### ELISA assay

Cell culture medium was recovered at every change of medium and was centrifuged at 13,000 rpm during 5 min. Supernatants were stored at − 80 °C until analysis. ELISA assay for interleukin-6 (IL-6) was performed with a specific human ELISA kit for IL-6 (Biosource Europe, Medgenix, Nivelles, Belgic) according to the manufacturer’s instructions.

### Metabolic assay

Cell growth was tested using WST-1 assay (Abcam, Cambridge, UK) attending the manufacturer’s recommendations at every medium change. Plates were read at 450 nm with a reference wavelength of 680 nm in an absorbance plate reader (Biotek).

### Viability calculation

Viability was tested using live/dead assay (Invitrogen) before and after detachment of the cultures following the manufacturer’s instructions. Moreover, viability calculation was performed using trypan blue exclusion assay on a Neubauer chamber after detachment of the cells with TrypLE Select® (Sigma-Aldrich).

### Statistical analysis

Experiments were performed in triplicate. A two-tailed Student’s *t* test was run, and *p* values < 0.05 were considered statistically significant (PRISM, version 6.0 GraphPad Software, San Diego, CA). Results are presented as the mean ± standard error (MD ± SE) or, in the case of the qPCR analysis, mean ± standard deviation (MD ± SD).

## Results

### XSHEM produced cells with LSC morphology and higher viability

**Fig. 1:**
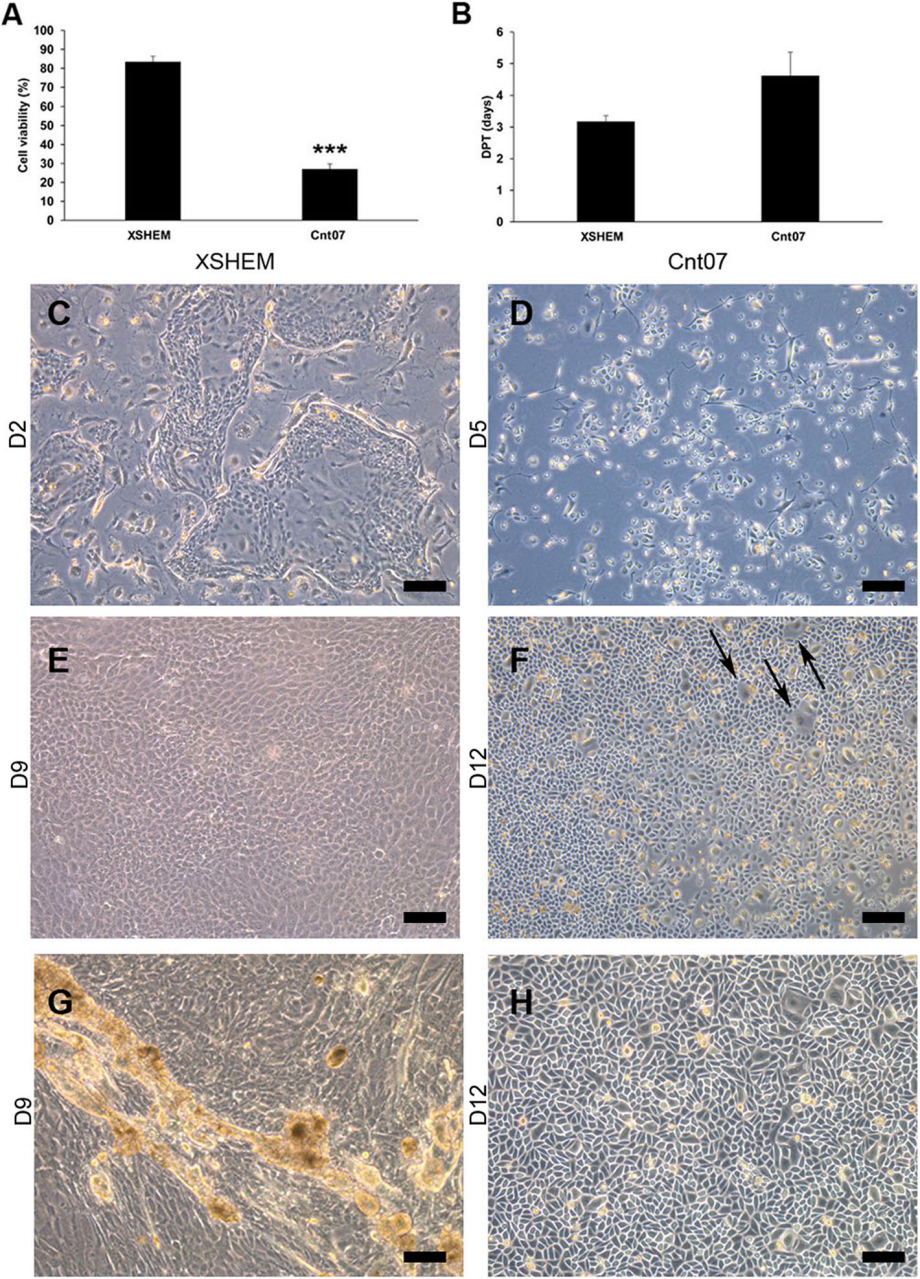
Comparison of the DPT, viability, and morphology of LSC grown with XSHEM or CnT07. **a** Evaluation of the viability by trypan blue exclusion assay. Cell viability was higher with XSHEM medium after cell detachment. **b** DPT did not show differences between both media. **c** Morphology of a starting culture of LSC cultured in XSHEM, corresponding to one clone in expansion. **d** Morphology of a starting culture of LSC cultured in CnT07. No clones could be observed. **e** Morphology of LSC cultured in XSHEM. Cells were small and polygonal. **f** Morphology of LSC cultured in CnT07. Cells had a cobblestone morphology, but differentiated cells could be observed (arrows). **g** XSHEM cultures showed stratifications as clones grew. **h** CnT07 cultures only grew forming monolayers and did not form any stratification. Results are presented as mean ± SE from three independent experiments. Statistical analysis was performed using two-tailed Student’s *t* tests (***p < 0.001). DPT, doubling population time; LSC, limbal stem cells; XSHEM, xenofree supplemented hormonal medium. Bar = 100 μm for **c** to **g**. Bar = 50 μm for **h**

We compared the culture features and the morphology of LSC when cultured with CnT07 and XSHEM medium. Moreover, we determined their doubling population time (DPT) and the viability at the end of the culture (Fig. 1). Cells cultured in CnT07 were small and cuboidal with a characteristic cobblestone morphology and grew forming a single monolayer (Fig. 1d, f). Usually, these cultures showed bigger cells with lower nucleus-to-cytoplasm ratio, interspersed between the smaller cells (Fig. 1h). Cells cultured with XSHEM were small and polygonal (Fig. 1e), and grew from colonies that rapidly merged forming thicker stratifications (Fig. 1c, e, g). At the end of the culture, before recombinant protease detachment, live/dead assay showed that cells had similar viability (Additional file 1: Figure S1). After recombinant protease detachment, cells cultured with CnT07 presented a more impaired viability than those in XSHEM medium, as showed by trypan blue exclusion assay (Fig. 1a) and by live/dead assay (Additional file 1: Figure S1). However, DPT did not show differences between both culture media (Fig. 1b).

### LSC in XSHEM and in CnT07 were positive for p63 and negative for corneal differentiation markers

**Fig. 2:**
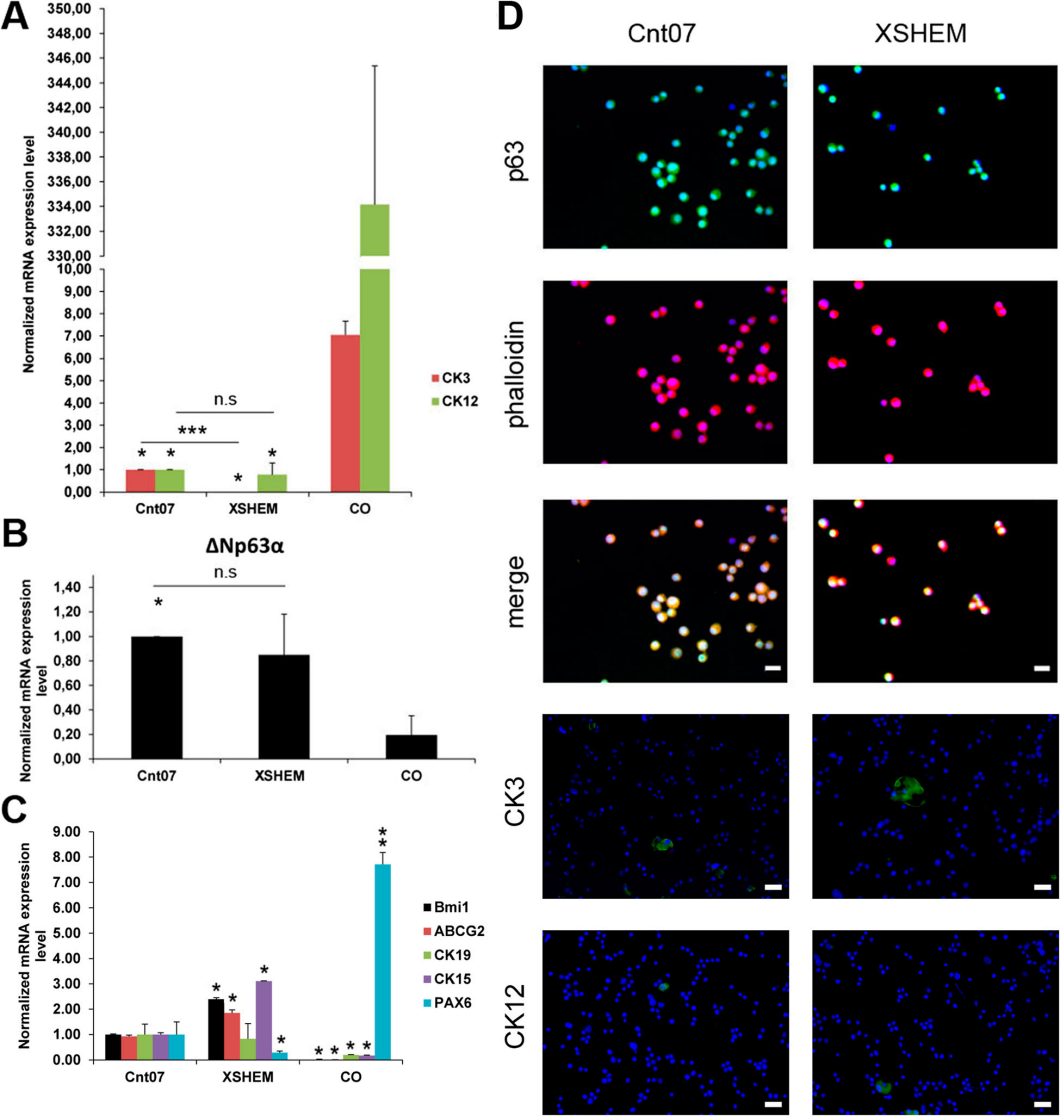
Expression of stemness and differentiation markers in LSC grown in XSHEM and CnT07. **a** Analysis of the mRNA expression levels for corneal differentiation markers CK3 and CK12. Their expression was minimal when compared with CO, although LSC cultured in CnT07 expressed more CK3 than those cultured in XSHEM. **b** Analysis of the mRNA expression levels for the stemness marker ΔNp63α did not show differences between LSC cultured in XSHEM or CnT07. **c** Analysis of the mRNA expression levels for Bmi1, ABCG2, CK19, CK15, and PAX6. **d** Immunofluorescence for p63, CK3, and CK12. Cultures showed a broad positivity for p63, while were widely negative for CK3 and CK12. Results are presented as mean ± SE from three independent experiments. Statistical analysis was performed using two-tailed Student’s *t* tests (*p < 0.05; ***p < 0.001; n.s, not significant). CK, cytokeratin; CO, human corneal epithelial cells; XSHEM, xenofree supplemented hormonal medium. Bar = 10 μm for p63. Bar = 25 μm for CK3 and CK12

We evaluated the expression of ΔNp63α, other characteristic stem cells, and corneal markers such as Bmi1, ABCG2, CK15, CK19, PAX6, CK12, and CK3, by qPCR and immunofluorescence (Fig. 2). Both XSHEM and CnT07 lead to LSC highly positive for ΔNp63α as demonstrated by immunofluorescence (Fig. 2d), with similar expression by qPCR (Fig. 2b). Meanwhile, the expression of Bmi1, ABCG2, and CK15 was higher in XSHEM (Fig. 2c). The mRNA expression of CK3, CK12, and PAX6 was negligible when compared to corneal epithelial cells (CO); the positive control obtained directly scrapped form central corneas (Fig. 2a, c). LSC cultured in CnT07 showed higher mRNA expression for CK3 and PAX6 than those cultured in XSHEM (Fig. 2a). However, both cultures were negative for CK3 and CK12 as assayed by immunofluorescence (Fig. 2d).

### PLA increased the clonogenicity and growth of LSC

**Fig. 3:**
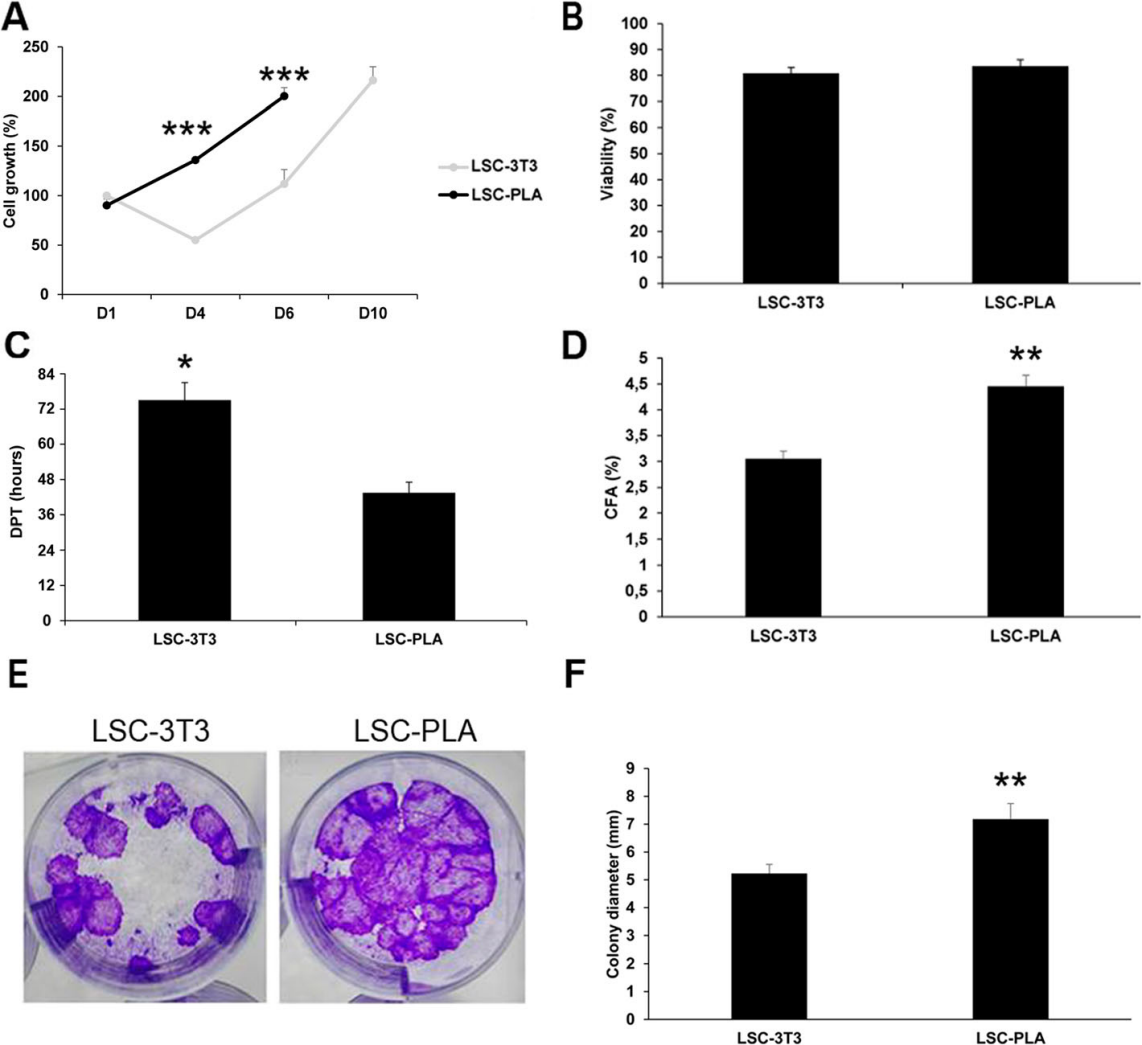
Cell growth, viability, and clonogenicity of the LSC cultured on murine 3T3 or human PLA feeder layers. **a** Cell growth evaluated by WST-1 was higher in LSC grown on PLA feeder layers during culture. **b** Evaluation of the LSC viability by trypan blue exclusion assay did not show differences after cell detachment. **c** DPT was higher in LSC cultured on PLA feeder cells. **d** Clonogenicity was increased in LSC cultured on PLA feeder cells (*n* = 6). **e** The colony diameters were larger in LSC cultured on PLA feeder cells. **f** CFA results are presented as mean ± SE from at least three independent experiments. Statistical analysis was performed using two-tailed Student’s *t* tests (**p < 0.01; ***p < 0.001). CFA, colony forming assay; D, day; DPT, doubling population time; LSC, limbal stem cells; LSC-3T3, co-cultures of LSC with 3T3 feeder layers; LSC-PLA, co-cultures of LSC with PLA feeder layers; PLA, processed lipoaspirate cells

Taking into account previous results, XSHEM medium was selected to assay the potential of PLA as feeder layers for LSC. To this end, we compared LSC cultured either with PLA or with 3T3, the gold standard feeder layer for CLET, for clonogenicity, DPT, and cell growth using WST-1 assay (Fig. 3). The LSC cultured on PLA feeder layer (LSC-PLA) took less time to reach sub-confluence than the LSC grown on 3T3 (LSC-3T3). Therefore, the DPTs were lower in these LSC (Fig. 3c), indicating faster cell growth. In fact, cell growth of LSC-PLA increased with culture time up to reaching sub-confluence at day 6 (D6). Instead, co-cultures of LSC-3T3 showed decreased cell growth at day 4 of culture (D4), and then, cell growth increased until the end of the culture at day 10 (D10) (Fig. 3a). Accordingly, LSC-PLA presented more ki67-positive cells at the end of the culture (Additional file 1: Figure S2), showed higher clonogenic capacity, and exhibited clones with bigger diameter (Fig. 3d, f). Moreover, viability tests by trypan blue assay and live/dead assay did not show differences after the protease detachment of the cultures (Fig. 3b and Additional file 1: Figure S1). No obvious cell death could be observed at the end of the cultures as well.

### LSC-PLA expressed more IL-6 and SDF-1 than LSC-3T3

**Fig. 4:**
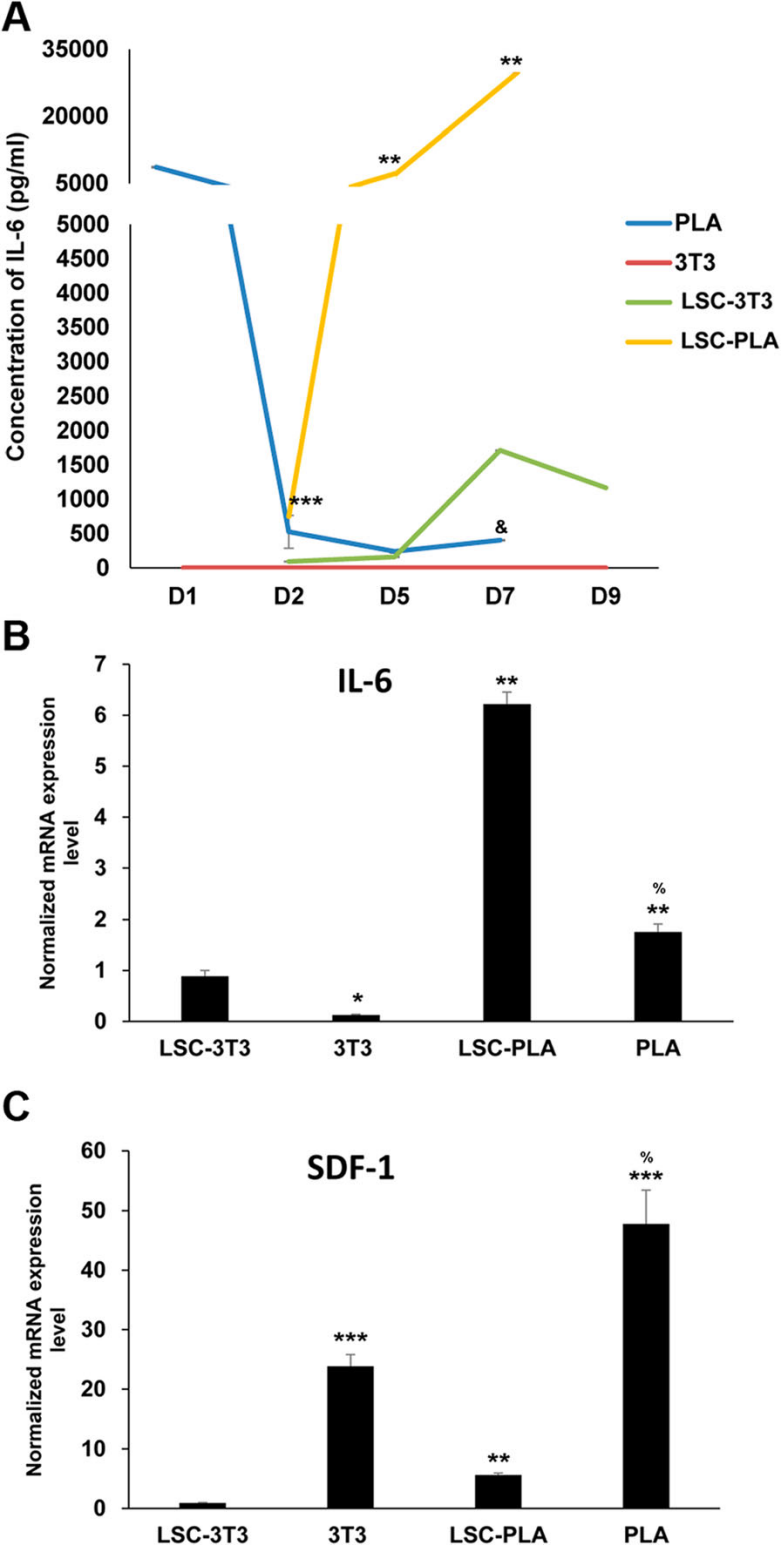
Expression of IL-6 and SDF-1. **a** ELISA assay detected increased expression of human IL-6 during the co-cultures of LSC with PLA. PLA monocultures also secreted higher levels of IL-6 during the first day of seeding. **b** Analysis of mRNA expression levels of IL-6 in 3T3 and PLA monocultures, and in LSC co-cultures with both feeder layers. **c** Analysis of mRNA expression levels of SDF-1 in 3T3 and PLA monocultures, and in LSC co-cultures with both feeder layers. Statistical analysis was performed using two-tailed Student’s *t* tests (**p < 0.01 compared to LSC-3T3; ***p < 0.001 compared to LSC-3T3; ^&^*p* < 0.001 compared to PLA; ^%^*p* < 0.01 compared to 3T3). D, day; IL-6, interleukin-6; LSC, limbal stem cells; LSC-3T3, co-cultures of LSC with 3T3 feeder layers; LSC-PLA, co-cultures of LSC with PLA feeder layers; PLA, processed lipoaspirate cells; SDF-1, stromal derived factor

To elucidate if the possible mechanism through PLA could favor the improvement of clonogenicity and cell growth of LSC, we analyzed the expression of IL-6 and SDF-1 (Fig. 4). The secretion of IL-6 was evaluated by specific human IL-6 ELISA assay at different culture time points in the supernatants of co-cultures and in the PLA and 3T3 cultures (Fig. 4a). Interestingly, at the first 24 h (D1), PLA feeders secreted their highest amount of IL-6. At day 2 (D2), the IL-6 levels decayed in these PLA cultures. At this time point, the initiated co-cultures of LSC-PLA started to increase their expression of IL-6. These levels of IL-6 secretion continued to increase remarkably until the end of the culture, at day 7 (D7). Conversely, PLA cultures retained lower levels of IL-6 until the end. LSC-3T3 secreted lower levels of IL-6 in comparison with LSC-PLA. IL-6 secretion by LSC-3T3 reached their maximum secretion at day 7 (D7), 2 days before reaching sub-confluence. As expected, due to the specificity for human IL-6, no IL-6 could be detected in the 3T3 cultures. So, a qPCR was run with specific primers for mouse IL-6 mRNA. The results showed that 3T3 cells expressed significantly lower mRNA expression than PLA (Fig. 3b). In addition, qPCR results for IL-6 confirmed ELISA data regarding LSC-PLA and LSC-3T3 co-cultures.

When assayed by qPCR, monocultures of PLA showed higher expression of SDF-1 than monocultures of 3T3 cells (Fig. 3c). Accordingly, the co-cultures of LSC and PLA showed a markedly increased expression of SDF-1 than the co-cultures of LSC and 3T3.

### Both PLA and 3T3 feeder generated cells with LSC characteristics

**Fig. 5:**
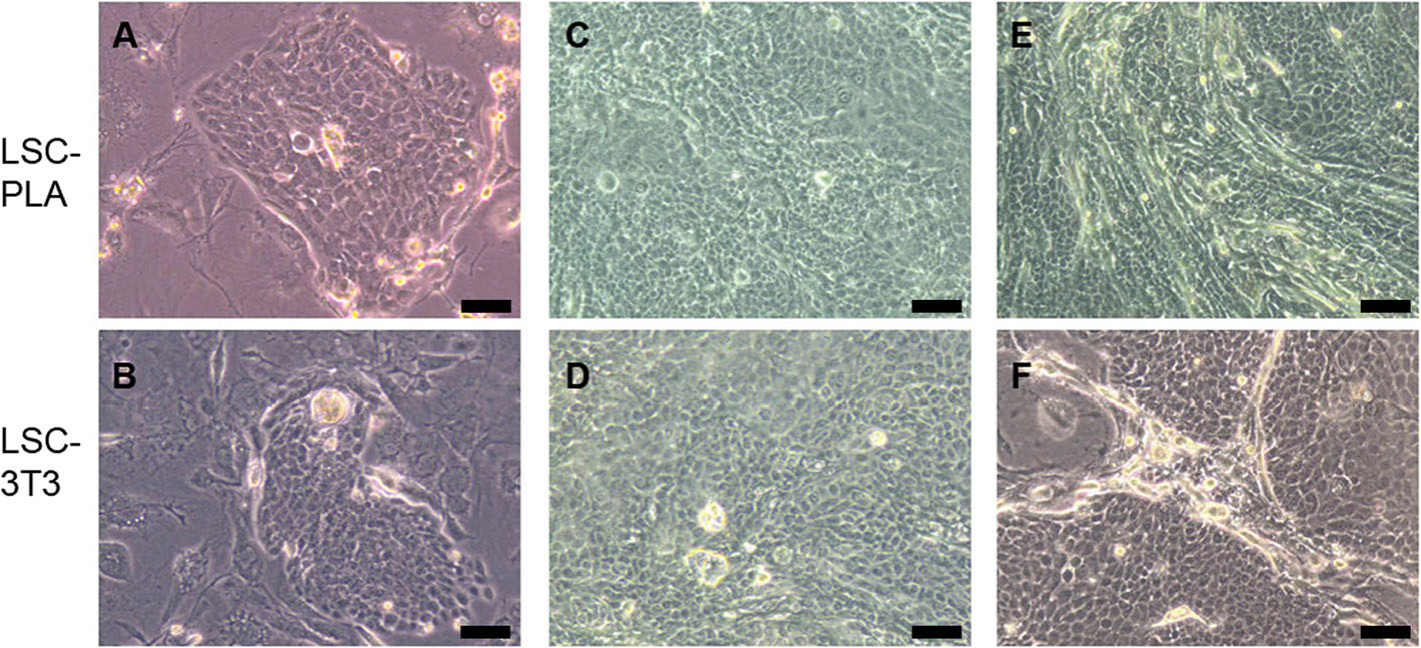
Morphology of LSC cultured on 3T3 or PLA feeder layers. **a**, **b** LSC cultured either on PLA or on 3T3 formed clones when the culture started. **c**, **d** In both cultures, cells were small and polygonal. **e**–**f** Clones of both cultures grew, merged, and formed stratifications. LSC, limbal stem cells; LSC-3T3, co-cultures of LSC with 3T3 feeder layers; LSC-PLA, co-cultures of LSC with PLA feeder layers; PLA, processed lipoaspirate cells. Bar = 50 μm for **a** and **b**. Bar = 100 for **c** to **f**

**Fig. 6:**
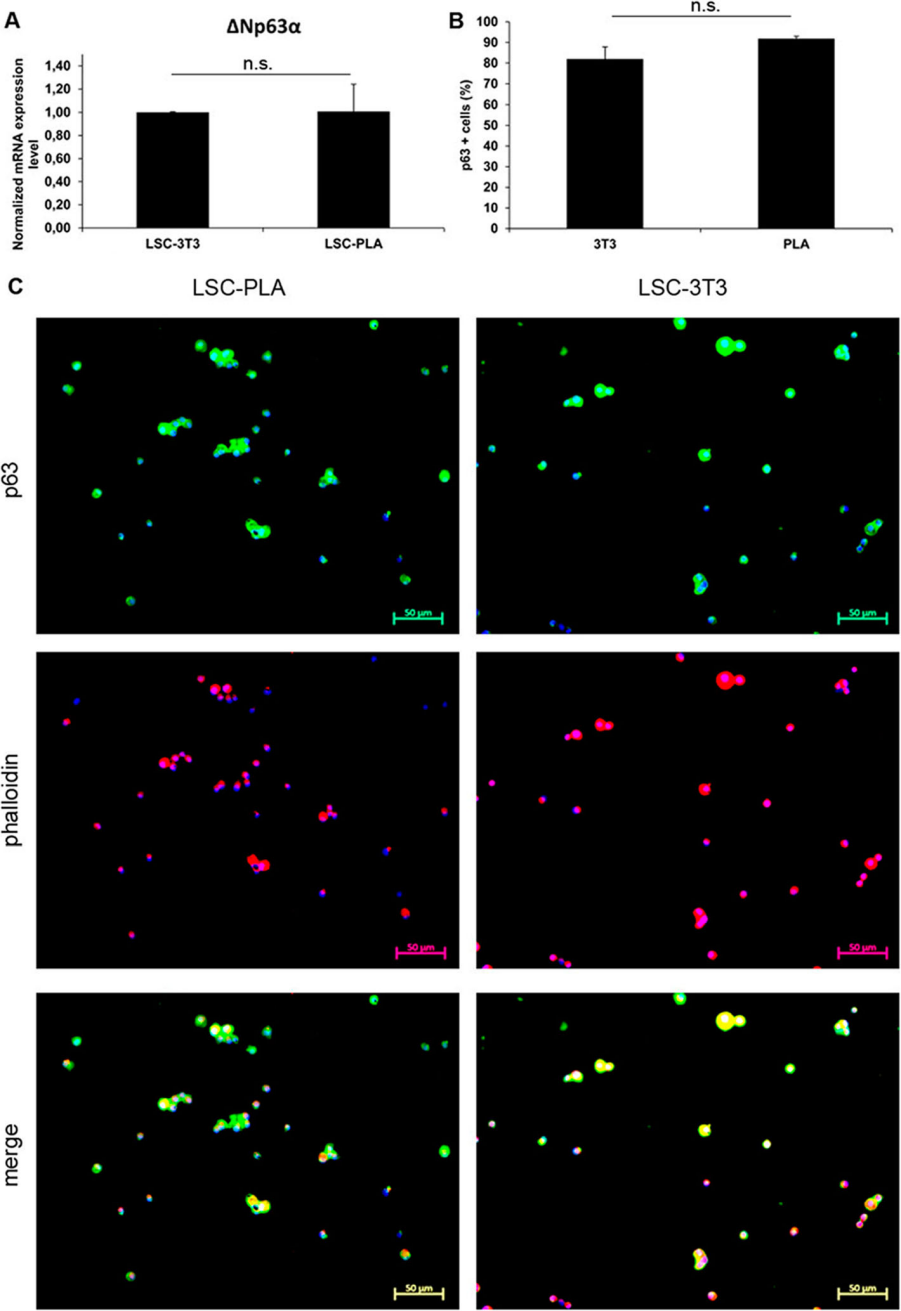
Expression of stemness and differentiation markers in LSC cultured with PLA and 3T3 feeder layers. **a** Analysis of the mRNA expression levels for stemness marker ΔNp63α. **b** The number (%) of p63-positive LSC cultured on PLA or 3T3 feeder layers did not show differences (*n* = 100). **c** Immunofluorescence quantification of p63-positive LSC cultured on PLA or 3T3 feeder layers did not show differences (*n* = 100). Immunofluorescence showing broad positivity for p63. Results are presented as mean ± SE from three independent experiments. Statistical analysis was performed using two-tailed Student’s *t* tests (*p < 0.05; **p < 0.01; ***p < 0.001; ns, not significant). CO, corneal epithelial cells; LSC, limbal stem cells; LSC-3T3, co-cultures of LSC with 3T3 feeder layers; LSC-PLA, co-cultures of LSC with PLA feeder layers; PLA, processed lipoaspirate cells. Bar = 50 μm

**Fig. 7:**
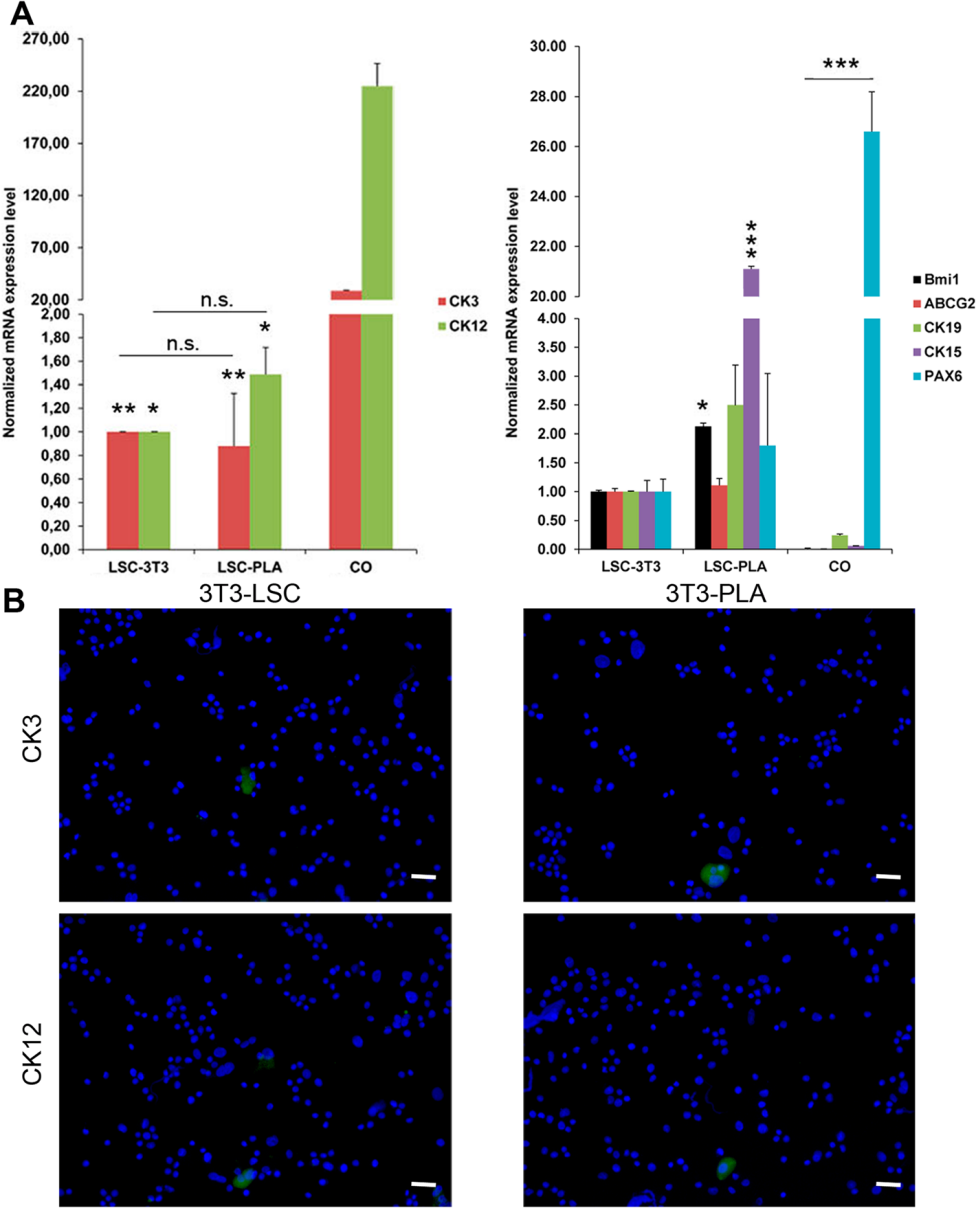
Expression of stemness and differentiation markers in LSC cultured with PLA and 3T3 feeder layers. **a** Analysis of the mRNA expression levels for CK3 and CK12 (corneal epithelial differentiation markers), and Bmi1, ABCG2, CK19, CK15 (putative LSC markers), and PAX6. There was minimal differences between LSC cultured on PLA or 3T3 feeder layers. **b** Immunofluorescence negative for CK12 and CK3. Results are presented as mean ± SE from three independent experiments. Statistical analysis was performed using two-tailed Student’s *t* tests (*p < 0.05; **p < 0.01; ***p < 0.001; ns, not significant). CK, cytokeratin; CO, corneal epithelial cells; LSC, limbal stem cells; LSC-3T3, co-cultures of LSC with 3T3 feeder layers; LSC-PLA, co-cultures of LSC with PLA feeder layers; PLA, processed lipoaspirate cells. Bar = 25 μm

Both LSC co-cultured on PLA and on 3T3 feeder layers were analyzed for their morphology and the expression of ΔNp63α, Bmi1, ABCG2, CK15, CK19, PAX6, CK3, and CK12 by qPCR and immunofluorescence. There were no differences regarding the morphology of LSC in both co-culture approaches; they were smaller, expanded from clones, and grew showing stratifications, until reaching sub-confluence (Fig. 5). Moreover, there were also no differences in the percentage of p63-positive cells in co-cultures, showing a broad positivity for ΔNp63α, as assayed by immunofluorescence (Fig. 6b, c). Results from qPCR corroborated similar mRNA expression for ΔNp63α between conditions (Fig. 6a). However, increased mRNA expression for Bmi1 and CK15 was detected in LSC-PLA without significant differences regarding ABCG2, CK19, and PAX6 expression (Fig. 7a). Negativity for CK3 and CK12 was observed by immunofluorescence and qPCR in LSC-PLA or LSC-3T3, and lower mRNA expression of PAX6 was detected in comparison with CO (Fig. 7a, b).

## Discussion

The search for clinical grade xenofree alternatives to culture LSC for advanced therapy is a need. Here, we demonstrated that LSC maintained an undifferentiated state and an adequate morphology, and improved its doubling population time and stemness when cultured in XSHEM on PLA feeder layer, without the intervention of any xenobiotic.

The maintenance of the size and the morphology of LSC have deep implications in their characterization [1, 2, 32]. LSC cultured with XSHEM media were smaller, with higher nucleus-to-cytoplasm ratio, and the cultures showed stratifications. Although stratification in LSC cultures is prevented in cell therapy because they can induce cell differentiation by cell confluence, this is a well-known trait of LSC cultures and an indication of the quality of the culture [33]. The cultures of CnT07 grew in monolayers, had an impaired viability after detachment, and had lower expression of progenitor markers Bmi1 and ABCG2. This pointed out that XSHEM maintains better LSC culture characteristics than CnT07, although there were no differences in the expression of the putative stemness marker p63 by qPCR and immunofluorescence. In addition, both medium conditions prevented the expression of corneal differentiation epithelial markers CK3 and CK12. Our results also support previous data showing that LSC express lower levels of PAX6 when compared to more differentiated progenies [34]. Moreover, our results are consistent with previous research showing that serum supplemented media maintain better survival and enrichment of LSC than commercial defined keratinocyte media [35].

The use of non-defined media has drawbacks, such as batch to batch serum variations [36]; however, “in house” formulations allow independence of commercial companies overcoming the need of further GMP validations if the product became discontinued. Another drawback of a non-defined medium is the potential risk of disease transmission [36]. However, this risk can also be managed effectively by the application of the European regulatory controls [17, 18]. Thus, microbiologic purity of the media can be tested and further validated to avoid disease transmission and assure an aseptic manufacturing process [37].

LSC growth under an undifferentiated state is proved with defined media only when supplemented with either human serum or synthetic supplements [20, 38]. Here, we showed that our defined medium without serum supplementation hinders the viability of LSC, making the culture less cost-effective. One explanation for the viability loss could be that the recombinant protease used for cell detachment was not completely inhibited by defined media, which usually contain low concentration of proteins and other elements such as calcium. Then, the cell viability loss in CLET could impair the outcomes of the transplantation by decreasing the survival of undifferentiated progenies [14]. For all these reasons, XSHEM medium represented a better alternative to culture LSC for human application and was chosen to test different feeder layers.

LSC cultures obtained in suspension systems directly on amniotic membrane without feeder layers in xenofree conditions produce grafts enriched in differentiated cells, positive for CK3 and CK12, indicating the loss of stem cell progenies [21]. This highlights the importance of the feeder layer to culture LSC. Although 3T3 feeder cells are necessary to maintain better cumulative LSC cell numbers even with xenofree medium [20], the risk of murine fibroblast use is the potential inflammatory responses against the graft generated by the presence of xeno-antigens in LSC transplant [39]. This would impair the results of the transplantation since any inflammatory reaction could lead to detrimental outcomes after grafting [40–43]. Avoiding this risk, one study changed the 3T3 feeder cells by human embryonic fibroblast cell line [22], demonstrating the same feasibility to support LSC cell growth. Since the use of human embryonic cells entails ethical implications and is related to teratogenic risks [44], somatic stem cell lines, such as PLA [45, 46], may be more advantageous for clinical applications. Human PLA are easy to obtain and isolate [45] and could be used for allogenic or autologous feeder layer purposes. Allogenic PLA feeder cells could be well characterized following international recommendations [25], screened for paracrine secretion, optimized to sustain LSC growth, and GMP-banked [46–48]. Moreover, PLA cells do not express HLA DRII [49] being invisible to the immune system and avoiding rejection in allogenic clinic application [50].

Successful clinical outcomes with CLET approach are related to the presence of undifferentiated progenies [14]. Since our culture system with XSHEM medium and PLA feeder layer generates undifferentiated LSC with higher clonogenic potential, expressing more Bmi1, highly positive for p63 and negative for the expression of CK3 and CK12, it is likely to provide better outcomes in clinical transplantation. Moreover, we demonstrated that PLA feeders induced faster proliferation and cell growth on LSC, so the cultures were more cost-effective. Moreover, the viability of LSC cultured on both feeders after detachment was similar. This fact makes sense, since both cultures used the same medium and the protease could be inhibited with the same efficacy.

Finally, there is a close interaction between LSC and their microenvironment, such as their neighboring cells and extracellular matrix, which regulate their proliferation and differentiation in the limbal niche [51]. When LSC are co-isolated with limbal stromal niche cells, the LSC proliferate faster and form more clones through the expression of SDF-1 [52], highlighting the importance of the communication between LSC and the stromal cells for their proliferation and maintenance. Moreover, IL-6 is secreted by limbal and stromal cells which act as a mediator between both cell populations, increasing the clonogenic potential and maintaining an undifferentiated state of LSC in in vitro culture systems [53]. Here, we also demonstrated that co-cultures with PLA induced more secretion of IL-6 and SDF-1, than co-cultures with 3T3, explaining the higher clonogenic capability, and the faster proliferation and cell growth. Human PLA secreted high amounts of IL-6 in the first 24 h of seeding, and its secretion could possibly influence the fate of LSC culture. In addition, it is well known that murine IL-6 does not have effect on human cells due to conformational differences within the IL-6 molecules [54]. This fact also supports the reasoning of using feeder layers from human origin to avoid species-specific effects.

## Conclusions

In summary, we demonstrated that the substitution of xenobiotics by human-derived alternatives is a feasible clinical grade xenofree option for LSC cultures in advanced therapy for ocular surface regeneration. The use of XSHEM medium combined with PLA feeder layers not only maintained LSC characteristics but also improved the LSC potential. This approach will have a direct clinical application in cell therapy for LSCD treatment.

## Supplementary information

**Additional file 1: Figure S1.** Live/dead assay for LSC cultured in all conditions before and after protease detachment. No obvious cell death could be observed at the end of the cultures. After detachment, cultures with Cnt07 medium showed impaired viability. Both conditions cultured with XSHEM, LSC-3T3 and LSC-PLA, did not showed differences in viability. Results are presented as mean ± SE from 3 independent experiments. LSC, limbal stem cells; LSC-3T3, co-cultures of LSC with 3T3 feeder layers; LSC-PLA, co-cultures of LSC with PLA feeder layers; PLA, processed lipoaspirate cells. Bar = 100 μm. **Figure S2.** Immunofluorescence for ki67 in LSC-3T3 and LSC-PLA. LSC-PLA showed higher percentage of ki67-positive cells than LSC-3T3 (*n* = 300). Results are presented as mean ± SE from 3 independent experiments. Statistical analysis was performed using two-tailed Student’s *t*-tests (^***^ *p* < 0.001). LSC, limbal stem cells; LSC-3T3, co-cultures of LSC with 3T3 feeder layers; LSC-PLA, co-cultures of LSC with PLA feeder layers; PLA, processed lipoaspirate cells. Bar = 25 μm.

**Additional file 2: Table S1.** Primary and secondary antibodies. **Table S2.** Primers and sequences.

## Data Availability

All data generated or analyzed during this study are included in this published article and its supplementary information tables and figures.

## Abbreviations

CK: Cytokeratin
CO: Human corneal epithelial cells
DPT: Doubling population time
IL-6: Interleukin-6
LSC: Limbal stem cells
LSC-3T3: Co-cultures of LSC with 3T3 feeder layers
LSC-PLA: Co-cultures of LSC with PLA feeder layers
PLA: Processed lipoaspirate cells
SDF-1: Stromal-derived factor
XSHEM: Xenofree supplemented hormonal medium
