# Supplementary figures and images for: Xenofree generation of limbal stem cells for ocular surface advanced cell therapy
Source: Stem Cell Res Ther. 2019 Dec 4;10:374. doi: 10.1186/s13287-019-1501-9 (PMC6894225; doi:10.1186/s13287-019-1501-9)

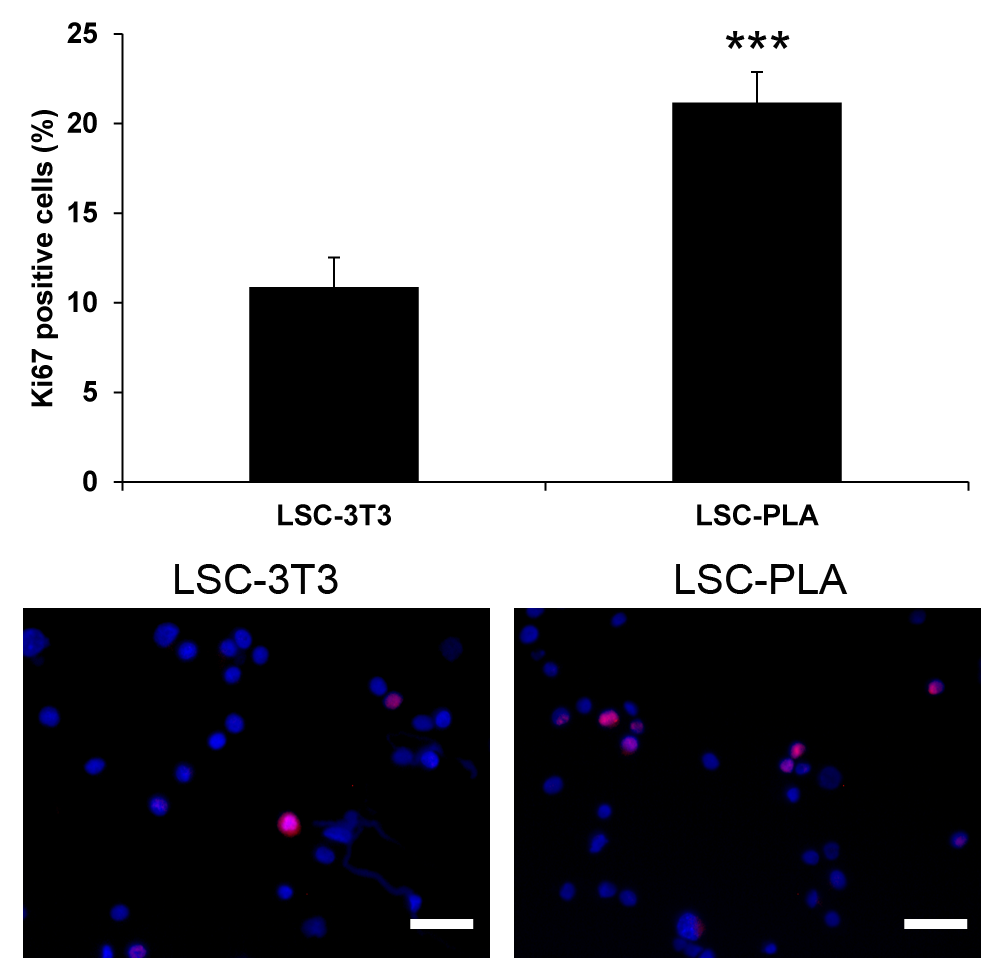

Supplement: Supplementary file 1 — Additional file 1: Figure S1. Live/dead assay for LSC cultured in all conditions before and after protease detachment. No obvious cell death could be observed at the end of the cultures. After detachment, cultures with Cnt07 medium showed impaired viability. Both conditions cultured with XSHEM, LSC-3T3 and LSC-PLA, did not showed differences in viability. Results are presented as mean ± SE from 3 independent experiments. LSC, limbal stem cells; LSC-3T3, co-cultures of LSC with 3T3 feeder layers; LSC-PLA, co-cultures of LSC with PLA feeder layers; PLA, processed lipoaspirate cells. Bar = 100 μm. Figure S2. Immunofluorescence for ki67 in LSC-3T3 and LSC-PLA. LSC-PLA showed higher percentage of ki67-positive cells than LSC-3T3 (n = 300). Results are presented as mean ± SE from 3 independent experiments. Statistical analysis was performed using two-tailed Student’s t-tests (*** p < 0.001). LSC, limbal stem cells; LSC-3T3, co-cultures of LSC with 3T3 feeder layers; LSC-PLA, co-cultures of LSC with PLA feeder layers; PLA, processed lipoaspirate cells. Bar = 25 μm. [file 13287_2019_1501_MOESM1_ESM.zip › FigureS2-EMMC.tif]

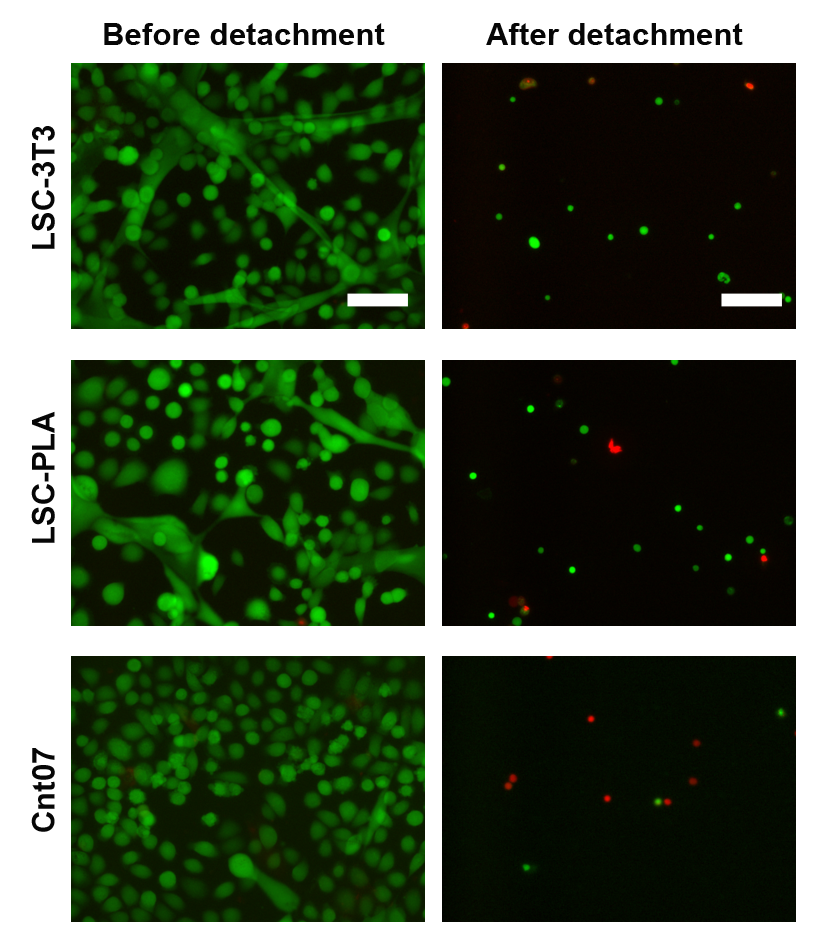

Supplement: Supplementary file 1 — Additional file 1: Figure S1. Live/dead assay for LSC cultured in all conditions before and after protease detachment. No obvious cell death could be observed at the end of the cultures. After detachment, cultures with Cnt07 medium showed impaired viability. Both conditions cultured with XSHEM, LSC-3T3 and LSC-PLA, did not showed differences in viability. Results are presented as mean ± SE from 3 independent experiments. LSC, limbal stem cells; LSC-3T3, co-cultures of LSC with 3T3 feeder layers; LSC-PLA, co-cultures of LSC with PLA feeder layers; PLA, processed lipoaspirate cells. Bar = 100 μm. Figure S2. Immunofluorescence for ki67 in LSC-3T3 and LSC-PLA. LSC-PLA showed higher percentage of ki67-positive cells than LSC-3T3 (n = 300). Results are presented as mean ± SE from 3 independent experiments. Statistical analysis was performed using two-tailed Student’s t-tests (*** p < 0.001). LSC, limbal stem cells; LSC-3T3, co-cultures of LSC with 3T3 feeder layers; LSC-PLA, co-cultures of LSC with PLA feeder layers; PLA, processed lipoaspirate cells. Bar = 25 μm. [file 13287_2019_1501_MOESM1_ESM.zip › FigureS1-EMMC.tif]
