# Supplementary material for: Xenofree generation of limbal stem cells for ocular surface advanced cell therapy
Source: Stem Cell Res Ther. 2019 Dec 4;10:374. doi: 10.1186/s13287-019-1501-9 (PMC6894225; doi:10.1186/s13287-019-1501-9)
Supplement: Supplementary file 2 — Additional file 2: Table S1. Primary and secondary antibodies. Table S2. Primers and sequences. [file 13287_2019_1501_MOESM2_ESM.zip › TableS2-SuppData-NietoNicolau.docx]

| **Table S2 (supplemental data).** Primers and sequences | | |  |
| --- | --- | --- | --- |
|  |  |  |  |
| **Gene** | **Forward (3'-5')** | **Reverse (5'-3')** | **Annealing** |
|  |  |  | **temperature** |
| ΔNp63α | GAAACGTACAGGCAACAGCA | GCTGCTGAGGGTTGATAAGC | 60°C |
| CK3 | GAGCGGCAACAGATCAAGAC | GGTAGCTCCGCAGGTAGTTG | 55°C |
| CK12 | TGGTCATGTTGGTCTTTGTAAC | ACTTCTCTCTATGCTCTTGACA | 55°C |
| IL-6 | CTGAGGCTCATTCTGCCCTC | AAGGCGCTTGTGGAGAAGG | 60°C |
| SDF-1 | GATTGTAGCCCGGCTGAAGA | TTCGGGTCAATGCACACTTGT | 60ºC |
| Mouse IL-6 | GAGGATACCACTCCCAACAGACC | AAGTGCATCATCGTTGTTCATACA | 60°C |
| RNA-18S | TATA center | TATA center | 60°C |
| Bmi1 | CCACCTGATGTGTGTGCTTTG | TTCAGTAGTGGTCTGGTCTTGT | 60ºC |
| ABCG2 | ACGAACGGATTAACAGGGTCA | CTCCAGACACACCACGGAT | 60C |
| CK19 | TGAGTGACATGCGAAGCCAAT | ACCTCCCGGTTCAATTCTTCA | 55ºC |
| CK15 | ACCACCACATTTCTGCAAACT | AGCTGAGATACTTCGGCTTCC | 55ºC |
| PAX6 | ATAACCTGCCTATGCAACCC | GGAACTTGAACTGGAACTGAC | 55ºC |
