# Supplementary material for: Xenofree generation of limbal stem cells for ocular surface advanced cell therapy
Source: Stem Cell Res Ther. 2019 Dec 4;10:374. doi: 10.1186/s13287-019-1501-9 (PMC6894225; doi:10.1186/s13287-019-1501-9)
Supplement: Supplementary file 2 — Additional file 2: Table S1. Primary and secondary antibodies. Table S2. Primers and sequences. [file 13287_2019_1501_MOESM2_ESM.zip › TableS1-SuppDat-NietoNicolau.docx]

|  |  |
| --- | --- |
| **Table S1 (supplemental data).** Primary and secondary antibodies | |
| **Antibody Source Isotype Clone Dilution** | |
|  |  |
| \| Cytokeratin 12 \| Abcam \| Rabbit IgG \| EPR1609(2) \| 1/100 \| \| --- \| --- \| --- \| --- \| --- \| \| Cytokeratin 3 \| Millipore \| Mouse IgG1 \| AE5 \| 1/100 \| \| p63 \| Abcam \| Mouse IgG2a \| BC4A4 \| 1/50 \| \| Ki67 \| Abcam \| Rabbit IgG \| ab15580 \| 1/1000 \| \| Phalloidin-TRITC \| Sigma \|  \|  \| 1/2000 \| \| Goat anti-rabbit 568 \| Sigma \| Goat \|  \| 1/2500 \| \| Rabbit anti-mouse 488 \| Sigma \| Rabbit \|  \| 1/2500 \| \| Goat A488 \| Invitrogen Life Technologies \| Goat \|  \| 1/1000 \| \| Mouse A488 \| Invitrogen Life Technologies \| Mouse \|  \| 1/1000 \| | |
|  | |
